# Single Nucleotide Polymorphisms Associated With Gut Homeostasis Influence Risk and Age-at-Onset of Parkinson's Disease

**DOI:** 10.3389/fnagi.2020.603849

**Published:** 2020-11-17

**Authors:** Anastazja M. Gorecki, Megan C. Bakeberg, Frances Theunissen, Jade E. Kenna, Madison E. Hoes, Abigail L. Pfaff, P. Anthony Akkari, Sarah A. Dunlop, Sulev Kõks, Frank L. Mastaglia, Ryan S. Anderton

**Affiliations:** ^1^Perron Institute for Neurological and Translational Science, Nedlands, WA, Australia; ^2^School of Biological Sciences, University of Western Australia, Crawley, WA, Australia; ^3^Centre for Neuromuscular and Neurological Disorders, University of Western Australia, Nedlands, WA, Australia; ^4^The Centre for Molecular Medicine and Innovative Therapeutics, Murdoch University, Murdoch, WA, Australia; ^5^Minderoo Foundation, Perth, WA, Australia; ^6^Institute for Health Research, University of Notre Dame Australia, Fremantle, WA, Australia; ^7^School of Health Sciences, University of Notre Dame Australia, Fremantle, WA, Australia

**Keywords:** Parkinson's disease, single nucleotide polymorphisms, gene-environment interactions, gut-brain axis, gut inflammation, toll-like receptors, peptidoglycan recognition protein

## Abstract

Research is increasingly focusing on gut inflammation as a contributor to Parkinson's disease (PD). Such gut inflammation is proposed to arise from a complex interaction between various genetic, environmental, and lifestyle factors, however these factors are under-characterized. This study investigated the association between PD and single-nucleotide polymorphisms (SNPs) in genes responsible for binding of bacterial metabolites and intestinal homeostasis, which have been implicated in intestinal infections or inflammatory bowel disease. A case-control analysis was performed utilizing the following cohorts: (i) patients from the Australian Parkinson's Disease Registry (APDR) (*n* = 212); (ii) a Caucasian subset of the Parkinson's Progression Markers Initiative (PPMI) cohort (*n* = 376); (iii) a combined control group (*n* = 404). The following SNPs were analyzed: *PGLYRP2* rs892145, *PGLYRP4* rs10888557, *TLR1* rs4833095, *TLR2* rs3804099, *TLR4* rs7873784, *CD14* rs2569190, *MUC1* rs4072037, *MUC2* rs11825977, *CLDN2* rs12008279 and rs12014762, and *CLDN4* rs8629. PD risk was significantly associated with *PGLYRP4* rs10888557 genotype in both cohorts. *PGLYRP2* rs892145 and *TLR1* rs4833095 were also associated with disease risk in the APDR cohort, and *TLR2* rs3804099 and *MUC2* rs11825977 genotypes in the PPMI cohort. Interactive risk effects between *PGLYRP2*/*PGLYRP4* and *PGLYRP4*/*TLR2* were evident in the APDR and PPMI cohorts, respectively. In the APDR cohort, the *PGLYRP4* GC genotype was significantly associated with age of symptom onset, independently of gender, toxin exposure or smoking status. This study demonstrates that genetic variation in the bacterial receptor *PGLYRP4* may modulate risk and age-of-onset in idiopathic PD, while variants in *PGLYRP2, TLR1/2*, and *MUC2* may also influence PD risk. Overall, this study provides evidence to support the role of dysregulated host-microbiome signaling and gut inflammation in PD, and further investigation of these SNPs and proteins may help identify people at risk of developing PD or increase understanding of early disease mechanisms.

## Introduction

Parkinson's disease (PD) is a debilitating neurodegenerative disorder with no cure. Although diagnosis and treatment typically center around motor impairments, PD is viewed as a complex, heterogeneous condition with a plethora of non-motor symptoms (Sauerbier et al., 2016; Ferreira and Massano, 2017). Notably, gastrointestinal dysfunction and the gut microbiome are increasingly studied in PD, both as disease symptoms and contributors to pathogenesis. For instance, many people with PD exhibit microbial dysbiosis (Bedarf et al., 2017; Hill-Burns et al., 2017; Gorecki et al., 2019) coupled with intestinal leakiness and inflammation (Forsyth et al., 2011; Devos et al., 2013; Clairembault et al., 2015; Schwiertz et al., 2018; Perez-Pardo et al., 2019) and future PD risk has been associated with antibiotic use (Mertsalmi et al., 2019), constipation (Adams-Carr et al., 2016), gastrointestinal infections (Nielsen et al., 2012; Nerius et al., 2019), and inflammatory bowel disease (IBD) (Villumsen et al., 2019). Furthermore, alpha-synuclein aggregation and phosphorylation throughout the enteric nervous system have been reported in people with PD (Braak et al., 2006) sometimes up to 20 years before PD diagnosis (Stokholm et al., 2016), but are not specific to PD (Visanji et al., 2015; Corbillé et al., 2016). Alpha-synuclein levels are increased throughout the enteric neurons of children with intestinal inflammation (Stolzenberg et al., 2017), adults with Crohn's disease (Prigent et al., 2019) and a high proportion of healthy individuals without a known neurodegenerative diagnosis during life (Gold et al., 2013), supporting a proposed role of alpha-synuclein in normal gut function and immunity (Barbut et al., 2019). However, truncal vagotomy reduces PD risk (Svensson et al., 2015), and rodent models support gut-to-brain transmission of alpha-synuclein aggregation via the vagus nerve in PD pathology (Kim et al., 2019; Challis et al., 2020). Consequently, the gut is implicated in early inflammatory processes that may contribute to alpha-synuclein aggregation and PD pathology in susceptible individuals (Hawkes et al., 2007; Houser and Tansey, 2017; Johnson et al., 2018), however the exact mechanisms are still unclear.

Environmental toxins (e.g., pesticides, herbicides, and heavy metals) and lifestyle factors (e.g., diet, smoking, and exercise) have been found to alter the gut microbiome and cause gut inflammation. These factors are associated with PD risk and progression, but are not always causative (Alcalay et al., 2012; Kamel et al., 2014; Fang et al., 2018; Angelopoulou et al., 2019; Marras et al., 2019). Furthermore, although several genetic mutations have been associated with autosomal dominant or recessive PD, disease presentation varies even among strongly penetrant forms of familial PD (Nishioka et al., 2006). Genome-wide association studies have identified 90 risk variants for sporadic PD (Nalls et al., 2019; Bandres-Ciga et al., 2020), though as yet they have not resulted in significant diagnostic or therapeutic translation. Notably, polymorphisms in inflammatory cytokines have previously been associated with PD risk (Hamza et al., 2011; Gao et al., 2012), and many genetic loci linked with PD risk are also associated with IBD (Lee et al., 2016; Rösler et al., 2018), a condition which also exhibits microbial dysbiosis and gut inflammation.

Rather than a single causative trigger, PD is proposed to arise from a complex interaction between various genetic, environmental and lifestyle factors which may contribute to chronic inflammation, oxidative stress, cytokine toxicity, alpha-synuclein aggregation, and eventual neuronal degeneration (Hawkes et al., 2007; Johnson et al., 2018). This is illustrated by comparison of human and rodent studies of *Pink1* mutations. In humans, *Pink1* mutations cause early-onset PD with almost 100% penetrance. Conversely, loss-of-function rodent models are healthy and do not develop characteristic PD-like symptoms, though intestinal infection with gram-negative bacteria in *Pink1* knockout mice causes PD-like symptoms, including an autoimmune response, dopaminergic neuronal alterations and motor impairments (Matheoud et al., 2019). Moreover, candidate gene or genome-wide association studies to investigate gene-environment interactions in PD have yielded mixed results (Dick et al., 2007; Hamza et al., 2011; Gao et al., 2012; Singh et al., 2014; Biernacka et al., 2016; Lee et al., 2016; Simon et al., 2017; Rösler et al., 2018), highlighting the complex nature of PD.

As the primary exchange site with the outside world, the gut is an important mediator of gene-environment interactions, especially through the gut microbiome and intestinal epithelium. Coupled with evidence linking gut inflammation to PD pathology, genetic variants that modulate intestinal integrity or microbiome-host signaling are an intriguing target for biomarkers or new therapeutic targets for PD and have so far received little attention. Consequently, the present novel study explored gut- and IBD-related single-nucleotide polymorphisms (SNPs) in subsets of two independent PD cohorts: the Australian Parkinson's Disease Registry (APDR) and the Parkinson's Progression Markers Initiative (PPMI). Specifically, the study investigated genetic polymorphisms in peptidoglycan recognition proteins *PGLYRP2* and *4*; toll-like receptors *TLR1, 2* and *4* and co-receptor *CD14*; tight-junction claudins *CLDN2* and *4*; and mucin glycoproteins *MUC1* and *MUC2*, to determine if they are associated with PD risk or age of symptom onset.

## Methodology

### Participants

#### APDR Cohort

This study utilized clinical data and samples from the Perron Institute for Neurological and Translational Science Biobank, obtained from 212 Caucasians with idiopathic PD from the Australian Parkinson's Disease Registry (APDR) who were sequentially recruited from Movement Disorders Clinics at the Perron Institute for Neurological and Translational Science (Perth, Australia) between 2007 and 2019, as previously described (Bakeberg et al., 2019). All participants reported no family history of PD, and were examined by a movement disorder neurologist prior to inclusion in the study for verification of the diagnosis in accordance with the UK Brain Bank criteria for idiopathic PD (Hughes et al., 1992). The use of these samples and data for the current study was approved by a Human Research and Ethics Committee (Approval number RA/2006/073 and RA/4/20/5308), and written informed consent was obtained from all participants in accordance with the National Health and Medical Research Council guidelines.

#### PPMI Cohort

This study drew upon 413 people with PD from the Parkinson's Progression Markers Initiative (PPMI) Cohort. Clinical information and sequencing data pertaining to target SNPs was obtained from the PPMI database (available at www.ppmi-info.org/data). SNPs genotypes were extracted from the available variant call format (VCF) files using rs identifiers with VCFtools (Danecek et al., 2011). All individuals categorized as Black, Asian, Other or Hispanic/Latino in the PPMI database were excluded from this study to create a Caucasian cohort which reflects the APDR group and avoids variation in gene frequencies arising from ethnicity.

#### Control Cohort

In order to consolidate control numbers, DNA and clinical data of healthy people from three separate databases was pooled. This consisted of 59 participants from the Perron Institute for Neurological and Translational Science Biobank; 179 from the PPMI cohort; and 166 age- and gender-matched healthy controls obtained from the NINDS Repository, Coriell Institute for Medical Research (New Jersey, USA). To minimize variability and to reflect the PD cohorts, only individuals categorized as Caucasian were included in this study. Minor genotype frequency, assessed through Levene's test, did not significantly differ between controls obtained from Australian, PPMI and Coriell cohorts (*p* > 0.05, data not shown) and so were combined into one healthy control cohort (*n* = 404) for subsequent analyses.

### Patient Information and Past Environmental Exposure

In addition to demographic and clinical information, APDR participants had previously reported information regarding pre-diagnosis smoking habits and toxin exposure, making this cohort enriched for information regarding environmental PD risk factors. Surveyed toxins included a variety of herbicides, pesticides, insecticides, fertilizers, heavy metals and industrial chemicals (summarized in Supplementary Table 1). Similar to previous research, participants were grouped as “Yes” for lifetime toxin exposure if they reported at least 7 days exposure prior to PD diagnosis (*n* = 99) or those reporting a main occupation in farming or agriculture (*n* = 14). Participants were grouped as “smokers” if they reported at least 6 months of regular cigarette smoking prior to their diagnosis (*n* = 91).

### DNA Extraction

For Australian participants, DNA was extracted from blood samples or buccal swabs. Blood was collected from medial cubital vein and DNA extraction occurred at respective state pathology services, being stored at −20°C until genotyping. Buccal samples were collected using Isohelix™ DNA/RNA Buccal Swabs (Cell Projects Ltd, Kent, U.K.) and stored in −20°C until DNA extraction using QIAamp DNA mini kits (Qiagen Pty LTD., Victoria, Australia).

### SNP Genotyping

SNPs previously associated with IBD and gastrointestinal integrity were investigated in this study, including immune signaling, microbial response or intestinal permeability (Table 1). APDR and Coriell samples were sent to the Australian Genome Research Facility (AGRF, Australia) for Agena Bioscience MassARRAY® genotyping on a Compact Spectrometer, conducted under blinded conditions. Assay design was conducted based on SNP sequence available from dbSNP (Supplementary Table 2). Genotyping data for PPMI cohort was obtained from the VCF files from whole genome sequencing data (available at www.ppmi-info.org/data).

**Table 1:** Single-nucleotide polymorphisms investigated in this study.

**Function**	**Gene**	**Chromosome**	**rs number**	**Location (Consequence)**
Pattern recognition receptors	Peptidoglycan recognition protein 2 (*PGLYRP2)*	19	rs892145	Exon (Missense)
	Peptidoglycan recognition protein 4 (*PGLYRP4)*	1	rs10888557	4KB upstream
	Toll like receptor 1 (*TLR1)*	4	rs4833095	Exon (Missense)
	Toll like receptor 2 (*TLR2)*	4	rs3804099	Exon (Synonymous)
	Toll like receptor 4 (*TLR4)*	9	rs4986790	Exon (Missense)
			rs7873784	3' UTR
	Cluster of differentiation 14 (*CD14)*	5	rs2569190	Intron
Intestinal barrier integrity	Mucin 1 (*MUC1)*	1	rs4072037	Exon (Synonymous)
	Mucin 2 (*MUC2)*	11	rs11825977	Exon (Missense)
	Claudin 2 (*CLDN2)*	X	rs12008279	Intron
			rs12014762^*^	3' UTR
	Claudin 4 (*CLDN4)*	7	rs8629	5' UTR

* *Variant spans CLDN2 and MORC4 genes*.

### Statistical Analysis

APDR and PPMI cohorts were separately compared to pooled controls using IBM-SPSS (v. 26, IBM Corporation). Significant nominal *p*-values of ≤ 0.05 (^*^) were employed. Normality was assessed utilizing Shapiro-Wilk test, and demographic information assessed through Mann-Whitney *U* or Chi-square tests where appropriate. When relevant, results were compared to the major genotype (underlined in all tables). To evaluate the association between SNPs and PD risk, a case-control logistic regression was performed in both a naïve (genetic variant only) and corrected models (genetic variant, gender, and age at assessment) comparing controls to APDR and controls to PPMI. Individuals were subsequently categorized by the presence of identified risk-increasing genotypes in order to calculate interactive risk effects through case-control logistic regression. Generalized linear models (GLMs) were used to investigate the association between SNPs and mean age of PD symptom onset in both cohorts (naïve model). All corrected GLM models include gender, but within PD analysis of the APDR cohort also incorporated information regarding environmental toxin exposures and lifetime smoking habits. Estimated means were calculated for significant SNPs from the GLMs, with pair-wise comparisons adjusted by Bonferroni correction for multiple comparisons.

## Results

### Cohort Information

Cohort demographics are summarized in Table 2. There was no significant difference in gender distribution between control, APDR and PPMI cohorts, however gender was included in all corrected models due to the presence of some X-linked SNPs. Mean age at assessment in the PPMI cohort (61.83 years ± 9.54, mean ± *SD*) was significantly lower than in the control (63.63 years ± 11.14) and APDR groups (65.80 years ± 8.99), and thus age at assessment was also included in subsequent corrected logistic regression models for risk. Finally, mean age of symptom onset for the PPMI cohort (59.91 years ± 9.71) was significantly later than the APDR group (57.63 years ± 10.10).

**Table 2:** Summary of cohort demographic information.

**Item**		**Control**	**APDR**	**PPMI**
*N*		404	212	376
Gender (%)	Male	65.1	62.7	65.7
	Female	34.9	37.3	34.3
Mean age (years)		63.6 (11.14)	65.8 (8.99)	61.8 (9.54)^*^^#^
Age of symptom onset (years)		-	57.63 (10.10)	59.91 (9.71)^#^

* *indicates p < 0.05 in comparison to controls*.

# *indicates p < 0.05 compared to APDR*.

### Influence of SNP Genotypes on PD Risk in APDR and PPMI Cohorts

Genotype frequencies of target SNPs and corrected regression models for PD risk in both APDR and PPMI are summarized in Table 3, with naïve regression models presented in Supplementary Table 3. Notably, heterozygotes of *PGLYRP4* rs10888557 demonstrated significantly increased risk of PD compared to the major GG genotype in both naïve [OR (95% CI) = 1.644 (1.070–2.526), *p* = 0.023] and corrected models [OR = 1.632 (1.057–2.520), *p* = 0.027] when comparing the APDR cohort to controls. Importantly, this finding was replicated when comparing the PPMI cohort to controls, as heterozygotes of rs10888557 also demonstrate significantly increased PD risk in both naïve [OR = 1.575 (1.085–2.288), *p* = 0.017] and corrected models [OR = 1.596 (1.095-2.327), *p* = 0.015].

**Table 3:** Corrected regression models evaluating the association between SNP genotype and disease risk in two PD cohorts.

**SNP**	**Genotype frequency (%)**				**Corrected regression models**			
					**APDR vs. C**		**PPMI vs. C**	
		**C**	**APDR**	**PPMI**	**OR (95% CI)**	***p***	**OR (95% CI)**	***p***
rs892145	AA	43.1	33.0	39.4	-	-	-	-
PGLYRP2	AT	46.0	**52.8**	47.3	**1.451 (1.006–2.093)**	0.046^*^	1.140 (0.842–1.543)	0.397
	TT	10.9	13.7	13.3	1.595 (0.917–2.770)	0.099	1.304 (0.821–2.071)	0.261
rs10888557	GG	84.2	77.4	78.2	-	-	-	-
PGLYRP4	GC	14.4	**21.7**	**21.0**	**1.632 (1.057–2.520)**	0.027^*^	**1.596 (1.095–2.327)**	0.015^*^
	CC	1.5	0.9	0.8	0.637 (0.126–3.211)	0.585	0.604 (0.149–2.444)	0.480
rs4833095	TT	56.7	62.7	57.7	-	-	-	-
TLR1	TC	36.4	34.4	36.7	0.864 (0.606–1.233)	0.421	0.998 (0.740–1.347)	0.991
	CC	6.9	**1.9**	5.6	**0.252 (0.086–0.737)**	0.012^*^	0.772 (0.424–1.404)	0.396
rs3804099	TT	33.2	35.8	25.5	-	-	-	-
TLR2	TC	49.3	47.6	**53.5**	0.900 (0.620–1.307)	0.581	**1.432 (1.031–1.990)**	0.032^*^
	CC	17.6	16.0	**21.0**	0.843 (0.512–1.391)	0.505	**1.553 (1.024–2.354)**	0.038^*^
rs4986790	AA	86.4	90.6	87.2	-	-	-	-
TLR4	AG	12.9	8.0	12.0	0.577 (0.323–1.031)	0.063	0.967 (0.629–1.487)	0.878
	GG	0.5	0.0	0.8	NA^#^	NA^#^	1.641 (0.271–9.951)	0.590
rs7873784	GG	71.0	72.2	68.4	-	-	-	-
TLR4	GC	26.7	22.2	29.3	0.836 (0.562–1.244)	0.377	1.133 (0.826–1.554)	0.439
	CC	2.0	4.2	2.4	1.994 (0.750–5.296)	0.166	1.276 (0.484–3.367)	0.623
rs2569190	GG	25.2	33.5	29.8	-	-	-	-
CD14	GA	52.7	44.8	49.2	0.683 (0.461–1.012)	0.057	0.790 (0.565–1.105)	0.168
	AA	22.0	21.7	21.0	0.758 (0.473–1.213)	0.248	0.817 (0.544–1.226)	0.328
rs4072037	TT	25.5	24.1	29.0	-	-	-	-
MUC1	TC	50.2	48.6	50.5	0.977 (0.645–1.480)	0.912	0.898 (0.642–1.257)	0.532
	CC	21.8	22.2	20.5	1.065 (0.651–1.743)	0.803	0.845 (0.561–1.274)	0.422
rs11825977	GG	62.1	62.3	68.9	-	-	-	-
MUC2	GA	34.7	33.5	**27.9**	0.948 (0.662–1.357)	0.770	**0.727 (0.534–0.990)**	0.043^*^
	AA	3.2	4.2	3.2	1.366 (0.562–3.318)	0.492	0.870 (0.388–1.951)	0.735
rs12008279	AA	50.2	47.2	44.9	-	-	-	-
CLDN2	AG	16.1	20.3	17.3	1.344 (0.766–2.360)	0.303	1.302 (0.803–2.112)	0.284
	GG	33.7	32.5	37.5	1.005 (0.687–1.469)	0.980	1.272 (0.928–1.741)	0.134
rs12014762	CC	78.0	70.8	75.3	-	-	-	-
CLDN2	CT	9.4	12.3	10.6	1.358 (0.744–2.476)	0.319	1.237 (0.730–2.096)	0.429
	TT	12.4	15.6	14.1	1.356 (0.830–2.215)	0.224	1.187 (0.775–1.818)	0.432
rs8629	CC	50.5	52.4	54.8	-	-	-	-
CLDN4	CT	40.6	37.3	39.1	0.883 (0.617–1.265)	0.497	0.888 (0.659–1.197)	0.437
	TT	8.9	9.9	6.1	1.076 (0.596–1.940)	0.809	0.644 (0.368–1.129)	0.124

*Major genotype is underlined. OR (95% CI), odds ratio (95% confidence interval)*.

* *indicates p < 0.05 for OR compared to major genotype*.

# *indicates unable to compute OR due to low sample number. Bold values indicate PD genotype groups which were significant in corrected regression compared to controls*.

Additionally, when analyzing the APDR cohort, *PGLYRP2* rs892145 AT heterozygotes demonstrated significantly higher PD risk than major AA homozygotes in both naïve [OR = 1.497 (1.041–2.152), *p* = 0.030] and corrected models [OR =1.451 (1.006–2.093), *p* = 0.046], with a greater but non-significant odds ratio with the minor TT genotype in naïve [OR = 1.638 (0.950–2.825), *p* = 0.076], and corrected models [OR = 1.595 (0.917–2.770), *p* = 0.071]. However, these results were not replicated in the PPMI cohort. Similarly, minor CC homozygotes of *TLR1* rs4833095 had significantly lower PD risk than major homozygotes in the APDR cohort in both naïve [OR = 0.246 (0.084–0.717), *p* = 0.010] and corrected models [OR = 0.252 (0.086–0.737), *p* = 0.012], but this was not replicated in the PPMI analysis.

Analysis between controls and PPMI demonstrated an increasing step-wise pattern for PD risk with *TLR2* rs3804099; TC heterozygotes exhibited significantly higher PD risk in naïve [OR = 1.410 (1.016–1.956), *p* = 0.040], and corrected regression [OR = 1.432 (1.031–1.990), *p* = 0.032]; a greater odds ratio was evident for minor homozygotes in naïve [1.553 (1.027–2.350), *p* = 0.037] and corrected models [OR = 1.553 (1.024–2.354), *p* = 0.038]. Finally, GA heterozygotes of *MUC2* rs11825977 had significantly reduced PD risk when comparing PPMI to controls in both naïve [OR = 0.727 (0.535–0.988), *p* = 0.042] and corrected regression models [OR = 0.727 (0.534–0.990), *p* = 0.043].

### Interactive Risk Effects of PGLYRP2, PGLYRP4, and TLR2 Genotypes

Additional regression models were computed to analyze the interactive effect of putative risk SNPs genotypes. When comparing APDR and controls, co-carriage of both *PGLYRP2* rs892145 AT and *PGLYRP4* rs10888557 GC significantly increased PD by two-fold compared to those without either risk genotype in naïve [OR = 2.030 (1.076–3.831), *p* = 0.029] and corrected models [OR = 2.016 (1.067–3.808), *p* = 0.031]. Similarly, when comparing PPMI and controls, co-carriage of both *PGLYRP4* rs10888557 GC and *TLR2* rs3804099 CC increased PD risk by ~1.8-fold but this was not significant in either naïve [OR = 1.835 (0.809–4.160), *p* = 0.146] or corrected models [OR = 1.846 (0.813–4.188), *p* = 0.143], likely due to the small number of individuals with both risk alleles in control (*n* = 10) or PD (*n* = 15). Subsequent analyses of *TLR2* rs3804099 heterozygotes with *PGLYRP4* rs10888557 GC genotype (control = 30, PD = 45) demonstrated significantly increased PD risk in naïve [OR = 1.883 (1.128–3.143), *p* = 0.015] and corrected models [OR = 1.890 (1.132–3.156), *p* = 0.015], suggesting an interactive allelic effect.

### PGLYRP4 rs10888557 SNP is Associated With Age of Symptom Onset in APDR Cohort

Among the APDR cohort (*n* = 212), 42.0% (*n* = 89), and 43.4% (*n* = 91) of participants reported lifetime toxin exposure and smoking, respectively. GLMs were computed to determine an association between SNPs and mean age of PD symptom onset, with results from three models displayed in Table 4: naïve, corrected for gender and reported lifetime toxin exposure (toxins) and corrected for gender and lifetime smoking status (smoking). *PGLYRP4* rs10888557 significantly predicted mean age of symptom onset in naïve (*p* = 0.029), toxin corrected (*p* = 0.029), and smoking corrected (*p* = 0.030) models. A step-wise pattern in age of symptom onset is evident upon pair-wise comparison of estimated means for rs10888557 genotypes in the naïve model, but did not withstand Bonferroni adjustment for multiple comparisons (*p*^adj^): GG homozygotes (56.70 ± 0.77 years) having significantly earlier age of symptom onset than heterozygotes (60.57 ± 1.46 years, *p* = 0.020, *p*^adj^ = 0.059), and minor CC homozygotes showing a non-significant step-wise trend (66.50 ± 7.01 years, *p* = 0.165, *p*^adj^ = 0.494). The significant difference between GG and GC genotypes was present in both corrected toxin (GC = 60.36 years ± 1.47, GG = 56.51 ± 0.82 years, *p* = 0.021, *p*^adj^ = 0.062) and smoking models (GC = 60.47 ± 1.46 years, GG = 56.67 ± 0.80 years, *p* = 0.021, *p*^adj^ = 0.058), but did not withstand Bonferroni correction. Overall, mean symptom onset was ~4 years later for the GC than the GG genotype. GLMs were repeated in the PPMI cohort, and there were no significant results in naïve models or after correction for gender (Supplementary Table 4).

**Table 4:** Generalized linear models investigating target SNPs and estimated mean age of PD symptom onset in enriched APDR cohort.

**Gene**	**SNP**	**GLMs in APDR (*****p*****-value)**		
		**Naïve**	**Corrected (Toxins)**	**Corrected (Smoking)**
PGLYRP2	rs892145	0.634	0.492	0.399
PGLYRP4	rs10888557	0.029^*^	0.029^*^	0.030^*^
TLR1	rs4833095	0.991	0.985	0.999
TLR2	rs3804099	0.934	0.928	0.887
TLR4	rs4986790	0.246	0.254	0.289
TLR4	rs7873784	0.663	0.666	0.747
CD14	rs2569190	0.187	0.146	0.153
MUC1	rs4072037	0.058	0.076	0.089
MUC2	rs11825977	0.792	0.652	0.648
CLDN2	rs12008279	0.971	0.489	0.531
CLDN2	rs12014762	0.490	0.626	0.615
CLDN4	rs8629	0.328	0.292	0.311

* p < 0.05 from test of model. Corrected (toxins), lifetime toxin exposure and gender; corrected (smoking), lifetime smoking status and gender. Bold values and *indicate p < 0.05 from test of model.

## Discussion

PD is increasingly considered the product of complex gene-environment interactions occurring in the gut, which are proposed to cause gastrointestinal, enteric and systemic inflammation and consequent PD pathology. Thus, it was hypothesized that a genetic predisposition for gut leakiness or inflammation, combined with environmental toxin exposure, may influence PD risk and symptom onset. Notably, this study reports a novel and robust association between PD risk and the *PGLYRP4* rs10888557 SNP in two independent cohorts of PD. In addition, rs10888557 genotype was determinant of the age of symptom onset in the APDR cohort, with carriage of the GC being associated with earlier age of onset. Furthermore, polymorphisms in other microbial pattern recognition receptors and one intestinal mucin were also associated with PD risk; where *PGLRYP2* rs892145 and *TLR1* rs4833095 were significant in the APDR cohort, while *TLR2* rs3804099 and *MUC2* rs11825977 were significant in the PPMI cohort. Moreover, previously unreported interactive effects of these SNPs on disease were demonstrated in both patient cohorts.

### Peptidoglycan Recognition Proteins

Notably, this study found an association between the *PGLYRP4* locus rs10888557 and PD risk as well as age of symptom onset. Expressed throughout the gut and secreted into the gut lumen, peptidoglycan recognition proteins (PGLYRPs) respond to peptidoglycans and other microbial products from gram-positive and gram-negative bacteria, exerting a bactericidal function to regulate gut homeostasis and cleaving peptidoglycan through amidase function (Dziarski and Gupta, 2010). In both the APDR and PPMI cohorts, *PGLYRP4* rs10888557 heterozygotes exhibited significantly increased PD risk compared to the major GG genotype. These results add to mixed literature concerning rs10888557 in PD, as CG and CC genotypes exerted a protective influence on PD risk among North Americans (Goldman et al., 2014), and no association was reported in a subsequent study in a northern Han Chinese cohort (Yu et al., 2018). The previously reported protective influence of the minor CC genotype on PD risk may contribute to the very low number of CC genotypes among people with PD and the consequent lack of statistical significance.

Coupled with the proposed role of the gut microbiome and complex-gene environment interactions in PD, these findings prompted further investigation regarding age of symptom onset incorporating self-reported exposure to environmental toxins and smoking history, factors known to influence PD risk (Kamel et al., 2014; Marras et al., 2019). In the APDR cohort, *PGLYRP4* rs10888557 GG homozygotes were found to have an ~4-years earlier age of symptom onset after controlling for both toxin exposure and smoking history, possibly indicating different underlying genetic mechanisms affecting disease risk and symptom time-course. However, these results were not replicated in the PPMI cohort in naïve or corrected models. Despite similar *PGLYRP4* genotype frequencies and risk associations in both cohorts, the PPMI has significantly later mean age of symptom onset than the Australian cohort and comes from a diverse international initiative. Thus, it is conceivable that associations between *PGLYRP4* genotype and disease onset are masked by the more culturally and environmentally heterogeneous nature of the PPMI cohort, and larger studies with more detailed analysis of gene-environment interactions are required.

Found upstream of *PGLYRP4* in a transcription factor binding site, publicly available data from the GTEx Portal (https://www.gtexportal.org/home/) indicate that rs10888557 is a significant expression quantitative trait locus (eQTL) for PGLYRP4 in various tissues, including the small intestine (terminal ileum). Additionally, variants in *PGLYRP4* have been associated with onset age of Crohn's disease (Zulfiqar et al., 2013), while rs10888557 has also been associated with ulcerative colitis risk in a Greek population (Gazouli et al., 2019). However, the effect of rs10888557 on *PGLYRP4* protein expression or function does not appear to have been studied. In light of the findings concerning PD risk and age of symptom onset in the current study, coupled with the notion that *PGLYRP4* variants may influence immune activity in response to microbial changes in the gut, further studies are required to investigate interactions between *PGLYRP4*, environmental toxins, the gut microbiome and PD pathology.

Additionally, it is important to acknowledge that the SNP rs10888557 is located between *PGLYRP4* and its neighboring gene *S100A9*. Data from NCBI (assembly GRCh38.p12) demonstrates that SNP rs10888557 is ~4,000 base pairs away from the 5' end of *PGLYRP4* and ~5,000 base pairs from the 5' end of *S100A9*. One of the S100 calcium-binding proteins, S100A9 is a pro-inflammatory mediator with amyloidogenic properties, and has been widely implicated in inflammation, cancer and neurodegeneration. Interestingly recent studies also demonstrate a specific role of S100A9 in PD, as S100A9 is elevated and co-localizes with alpha-synuclein in PD brains *ex vivo*, and induces alpha-synuclein aggregation *in vitro* (Horvath et al., 2018). Interestingly, S100A8 and S100A9 dimers create calprotectin, where elevated fecal calprotectin levels indicate increased neutrophil migration in response to gut barrier dysfunction. Such elevated fecal calprotectin levels are evident in people with PD (Schwiertz et al., 2018; Mulak et al., 2019), and it is hypothesized that the amyloidogenic properties of calprotectin and S100A9 may contribute to fibril formation and enteric alpha-synuclein aggregation in response to gut inflammation (Mulak et al., 2019). Given the link between S100A9 and PD, further studies should investigate if rs10888557 affects *S100A9* transcription factor binding or is a marker for regional variation not limited to the *PGLYRP4* gene.

Found in the same peptidoglycan recognition receptor family as *PGLYRP4*, rs892145 in *PGLYRP2* was also associated with PD risk in the APDR cohort, where heterozygotes had significantly increased risk of PD, with a similar non-significant trend evident for minor TT homozygotes in comparison to major homozygotes. This echoes a previous study demonstrating reduced PD risk with AT and AA genotypes in a North American population (Goldman et al., 2014), but was not replicated in the predominately North American PPMI PD cohort in the current study, highlighting the need for further investigation. The missense variant rs892145 is an eQTL in whole blood and liver, suggesting a possible role in modulating PGLYRP2 response to circulating pathogens arising from a leaky gut. Additionally, the expression of PGLYRP2 is induced in oral epithelial cells after bacterial exposure (Scholz et al., 2018), and thus a missense variant may influence downstream immune responses in other tissues.

### TLRs

TLR1-TLR2 heterodimers recognize triacylated microbial lipoproteins from gram-negative bacteria and mycoplasma to cause an innate immune response (Burgueño and Abreu, 2020), and variants in *TLR1* and *TLR2* were associated with PD risk in the APDR and PPMI cohorts, respectively. Regarding *TLR1* rs4833095, the CC genotype and C allele are known to be associated with increased risk of Crohn's disease and ulcerative colitis (Bank et al., 2014), and increased risk of *Helicobacter pylori* infection in Chinese (Yang et al., 2013) and Thai populations, with worse mucosal inflammation and altered morphology after infection in the latter (Tongtawee et al., 2016). These conditions and infections are associated with increased future PD risk (Nielsen et al., 2012; Villumsen et al., 2019). Additionally, GTEx Portal reports that rs4833095 is a significant eQTL for TLR1 in various tissues, including the colon. Contrastingly, the current study reports significantly reduced PD risk with *TLR1* rs4833095 CC genotype in the APDR cohort. The minor C allele of rs4833095 impairs TLR1 signaling and subsequent inflammatory cascades (Omueti et al., 2007), while the major T allele is linked with higher levels of interleukins IL-12p40 and IL-17 (Santana et al., 2017). Thus, further studies are required to assess if an impaired TLR1 response facilitates reduced resistance to microbial pathogens, which may lead to future infection, chronic inflammation or PD pathology in the long term.

The current study demonstrates that heterozygotes and minor CC homozygotes of *TLR2* rs3804099 had significantly increased PD risk in the PPMI cohort compared to healthy controls, supporting a link between infections, altered TLR2 signaling, chronic inflammation and PD. However, in contrast to the largely North American and European Caucasian PPMI cohort, the major TT allele was previously associated with increased PD risk among North-eastern Han Chinese especially among those with late-onset PD (Li et al., 2017), indicating possible geographical, cultural or ethnic differences mediating the link between TLR2 and PD pathology. The synonymous *TLR2* variant rs3804099 is a significant eQTL for cultured fibroblasts, brain and esophagus tissue on GTEx, and was previously associated with susceptibility to *H. pylori* infection and gastric cancer pathogenesis (Mirkamandar et al., 2018; de Matos Lourenço et al., 2020). Specifically, CT and TT genotypes of rs3804099 are linked with resistance to bacterial infection due to higher TNF-a, IL-1B and IL-6 production in peripheral blood monocytes than CC homozygotes (Zhang et al., 2013). Given that TLR1 expression and TLR1/TLR2 function exhibit an age-related decline (Van Duin et al., 2007), coupled with a lack of validation for *TLR1* rs4833095 and *TLR2* rs3804099 in both studied cohorts, further investigations of larger cohorts are required to clarify the relationship between TLR1, TLR2, and PD risk.

Finally, heterozygotes of *TLR4* rs4986790 and *CD14* rs2569190 demonstrated markedly reduced PD risk [odds ratio = 0.577 (*p* = 0.063) and 0.683 (0.057), respectively], where a lack of significance may be due to the sample size of the cohort. Membrane-associated CD14 facilitates binding of LPS by TLR4, and co-localization of CD14 and TLR4 are necessary for LPS-mediated alterations to epithelial tight-junction proteins (Guo et al., 2013). Given increasing evidence concerning a role of LPS and TLR4 in PD (Gorecki et al., 2019, 2020; Perez-Pardo et al., 2019), further targeted studies investigating TLR4 and CD14 polymorphisms in larger cohorts are warranted.

### Mucin

The current study reported reduced PD risk among *MUC2* rs11825977 heterozygotes in the PPMI cohort. MUC2 is a highly glycosylated mucin secreted by goblet cells which contributes to the two mucosal layers overlying the intestinal epithelium, and is highly expressed throughout the small intestine and colon (Moehle et al., 2006). Both these mucosal layers are the first line of innate host defense to intestinal pathogens, and the outer layer is home to commensal microbes (Johansson et al., 2011). Importantly, MUC2 expression is reduced in IBD in humans, and MUC2 deficiency causes intestinal inflammation, spontaneous colitis (Van der Sluis et al., 2006) and gut dysbiosis in mice (Wu et al., 2018). Although not a significant eQTL on GTEx, the missense variant 11825977 (also known as V116M) has been linked to reduced mRNA expression of *MUC2* and increased risk of Crohn's disease (Moehle et al., 2006). It is conceivable that rs11825977 influences intestinal inflammation and gut dysbiosis which could subsequently contribute to PD onset.

### Limitations

The present study has a number of limitations. Firstly, as ethnicity can influence the immune system and inflammatory conditions such as IBD (Nahid et al., 2018; Gazouli et al., 2019), the study only included individuals categorized as Caucasian. However, significant genetic and cultural variation exists between Caucasians from Australia, North America and Europe. This may influence not only allele frequencies, but also environmental and lifestyle factors (e.g., occupation and diet) that in turn could alter gene expression and function (Liu et al., 2020), likely contributing to the discrepancies evident between cohorts in the current study. Further studies in larger multi-ethnic populations will therefore be important. Another limitation of the present study is the retrospective and self-report nature of toxin exposure data, which was restricted to people with PD in the APDR cohort. While the novel and robust finding of an association between *PGLRYP4* polymorphism and age of symptom onset among Australians with PD was not altered when controlling for toxin exposure or lifetime smoking habits, further studies with more comprehensive information for both the PD and healthy control group may yield further significant findings. Moreover, recent studies demonstrate the importance of considering other forms of genetic variations in such association studies, particularly in the context of complex diseases such as PD (Theunissen et al., 2020). Previous studies also report that genetic variants may have regional effects beyond a single gene, so further studies of rs10888557 loci in the context of PD should include an analysis of other SNPs and structural variations (i.e., in non-coding regions) within the region of *PGLYRP4* and *S100A9* genes in order to investigate and potentially establish the extent of association (Roses et al., 2016; Theunissen et al., 2020).

### Future Directions

Overall, the current study reports novel associations between PD and SNPs involved with gut homeostasis and immune signaling, supporting previous literature concerning gut inflammation and altered host-microbiome signaling in PD. For instance, studies combining microbial and metabolomic profiling demonstrate reduced levels of short-chain fatty acids and associated short-chain fatty acid-producing bacteria, which typically exert an anti-inflammatory influence (Unger et al., 2016; Vascellari et al., 2020). Moving forward, investigating the interaction between genes, environment and the gut microbiome through combination of genetic sequencing, microbial and metabolomics profiling may provide novel insights into the mechanisms of gut inflammation in PD, identify specific biomarkers for earlier diagnosis prior to significant neurodegeneration, and facilitate precision medicine for better disease management. Notably, genetic stratification by the gut-related SNPs identified in the current study may increase the efficacy of current gut-targeted therapeutic options for PD. For example, probiotic administration has shown promise in alleviating PD symptoms in both rodent models and people with PD (Barichella et al., 2016; Dutta et al., 2019; Tamtaji et al., 2019), and probiotic administration was particularly beneficial among children carrying high-risk SNPs for eczema (Morgan et al., 2014). Moreover, the anti-inflammatory and gut barrier-promoting effects of probiotic administration are attributed to TLR2-dependent mechanisms (Kuugbee et al., 2016; Paveljšek et al., 2020), and thus further studies are required to investigate if probiotic administration to people with pro-inflammatory TLR2 SNPs has enhanced benefit for PD symptoms. Lastly, investigation of these and other gut-related SNPs may be beneficial for other complex neurodegenerative disorders which are also associated with gut dysfunction and dysbiosis, including Alzheimer's disease, Huntington's disease, and multiple sclerosis.

### Conclusion

This study aimed to elucidate risk and disease-modifying effects of anti-microbial and intestinal-related polymorphisms in PD. Importantly, we report robust significant associations between *PGLYRP4* and disease risk in two independent PD cohorts, and a novel association between *PGLYRP4* and age of symptom onset in an Australian PD cohort, as well as interactive risk-modifying effects of polymorphisms in the *PGLYRP2, PGLRYP4*, and *TLR2* genes. The various SNPs which were associated with risk in the current study may confer protection from or susceptibility to complex inflammatory processes, and the mixed results evident between cohorts highlight the need for larger, prospective studies investigating genetic predispositions, environmental exposures, and lifestyles factors to examine the multifaceted nature of PD. In light of increasing evidence concerning the involvement of the gut microbiome and immune system in PD, further studies are required to investigate the functional implications of the significant variants identified in this study, which may contribute to, or exacerbate, PD pathogenesis and pathology upon interaction with other factors.

## Data Availability Statement

The raw data supporting the conclusions of this article will be made available by the authors, without undue reservation.

## Ethics Statement

The studies involving human participants were reviewed and approved by University of Western Australia Human Research and Ethics Committee. The patients/participants provided their written informed consent to participate in this study.

## Author Contributions

AG, SD, FM, and RA: study design. MB, JK, and FM: clinical data and genetic sample collection. AG, MB, FT, MH, and PA: APDR and control DNA extraction and SNP analysis. AG, AP, and SK: PPMI data analysis. AG, MB, FT, FM, and RA: statistical analysis. AG: manuscript draft. AG, MB, SD, FM, and RA: critical revision. All authors contributed to the article and approved the submitted version.

## Conflict of Interest

The authors declare that the research was conducted in the absence of any commercial or financial relationships that could be construed as a potential conflict of interest.
