# Supplementary material for: Single Nucleotide Polymorphisms Associated With Gut Homeostasis Influence Risk and Age-at-Onset of Parkinson's Disease
Source: Front Aging Neurosci. 2020 Nov 17;12:603849. doi: 10.3389/fnagi.2020.603849 (PMC7718032; doi:10.3389/fnagi.2020.603849)
Supplement: Supplementary file 1 [file Table_1.docx]

Supplementary Material

**Supplementary Table 1.** Reported toxin exposure in enriched APDR cohort

| **Type** | **Names** | ***N* ever exposed** |
| --- | --- | --- |
| Acid | Citric Acid | 1 |
|  | Hydrochloric Acid | 10 |
| Fertilizers | Containing phosphoric acid/phosphate salts | 1 |
|  | Containing sulphuric acid | 2 |
| Fungicide | 245T (2,4,5-Trichlorophenoxyacetic acid) | 1 |
|  | 24D (2,4-Dichlorophenoxyacetic acid) | 3 |
|  | Dicamba (3,6-dichloro-2-methoxybenzoic acid) | 1 |
|  | Fenoprop/245 TP | 1 |
|  | Hexachlorophene | 1 |
|  | MCPA (2-methyl-4-chlorophenoxyacetic acid) | 1 |
|  | Round-up (Glyphosate) | 4 |
|  | Sprayseed | 2 |
|  | Dieldrin | 7 |
| Insecticide | Lindane (Gamaxine) | 2 |
|  | Malathione | 4 |
| Pesticide | 1080 (Sodium fluroacetate) | 2 |
|  | Baygon/Dipterex | 1 |
|  | DDT (Dichlorodiphenyltrichloroethane) | 10 |
|  | Misc. | 4 |
|  | Rotenone | 1 |
| Industrial chemicals | Arsenic | 5 |
|  | Benzene | 1 |
|  | Bromide | 1 |
|  | Chlorine | 1 |
|  | Cyanide | 4 |
|  | DIBK (Disobutyl Ketone) | 1 |
|  | Formaldehyde& formalin | 4 |
|  | Industrial Glue | 1 |
|  | Misc. painting chemicals | 3 |
|  | Sodium hydroxide, lye, Caustic Acid | 4 |
|  | Solder flux fumes | 1 |
|  | Welding chemicals | 6 |
| Misc. Chemicals | Ammonia | 2 |
|  | Asbestos | 1 |
|  | Carbon Tetrachloride | 1 |
|  | Copper Sulphates | 1 |
|  | Hydrocarbons | 1 |
|  | Mercury | 3 |

**Supplementary Table 2.** SNP sequence used for genotyping of Australian and Coriell samples

| **SNP** | **Sequence** |
| --- | --- |
| rs1884444 | TTATGCTTTTTATTATTTTACTAAAATACTACAATTTAAACATTTTTCATATTTTTTTTCCAGAGGGAAACAGTCTTTTCCTGCTTCCAGACATGAATCA[G/T]GYCACTATTCAATGGGATGCAGTAATAGCCCTTTACATACTCTTCAGCTGGTGTCATGGAGGTATGGTGTTTTATGTATCTATTGCTATCTTTCATTCAA |
| rs11209026 | TTAGACAACAGAGGAGACATTGGACTTTTATTGGGAATGATCGTCTTTGCTGTTATGTTGTCAATTCTTTCTTTGATTGGGATATTTAACAGATCATTCC[G/A]AACTGGGTAGGTTTTTGCAGAATTTYTGTTTTCTGATTTAGACTACATGTATATGTATCACCAAAATTTAGTCATTTCAGTTGTTTACTAGAAAAATCTG |
| rs4149570 | CTGGGAGTCAAATTCTCTGGGTTCCAATTCAGAATGCTTAGCTTTTTAGCTAAGAATGTGTCTTGGACACAYAAATGAACTTCTCAGACACATAACTGAA[A/C]CTGTCTGGATCTGTTTTCCAATTTGCAGAGTGGAGATAACCTGTCTCCTGGGGTTCCTGTAAGGATTTGTTCAGATGGGAGAGATATAGATAAGCGTCCG |
| rs892145 | GTACTGGCTCAGCAAGTGGCTGAGGGATGGCCGGGGCTCAGGAGTCCGGCTCAGGTAGTCTCCAAGGATGACCCCATCCAGGGCGCCATTGAGGAAGGCC[A/T]TGGTTAACAGAGATGCCTTGGGGTCCAAAAGCGTAAAGKTCCGAGGGGCAGAGAGCTGGTCCCAGCAGCCCTCAGTTCCCAGGTCTGGATGGCTCTGGGT |
| rs10888557 | TAAATRCTGATACATCTAATCCACAAAAGCTCTTTGGGATACTCAATAACTTCTATGAATACATATAAAAGGGTCCAGAGAGCAAAAAGATTAAAAACCT[G/C]GGGCCAAAGGAATCCCACAGGCTGTCCTGATGCAGATAGGCCACAAGGTGGCAGCATGCGCCCTCTGATAACTCCGGAGYCACCTTTCACTCACTAGGAG |
| rs4833095 | CACGTTTGAAATTGAGAAATACCATACAGTTGTATGCCAAACCAGCTGGAGGATCCTAATGAAAGAATTCCAAGTTGTTTCAATGTTGTTTAAGGTAAGA[T/C]TTGATAACTTTGGATTTGTTTGAAGTTTCGCCAGAATACTTAGGAAGTAAGAACATTTGTTATCTTCTAGCACACATTTGATATTAGATAGTTCCAGATT |
| rs3804099 | AAAGAAAAGATTTTGCTGGACTTACCTTCCTTGAGGAACTTGAGATTGATGCTTCAGATCTACAGAGCTATGAGCCAAAAAGTTTGAAGTCAATTCAGAA[T/C]GTAAGTCATCTGATCCTTCATATGAAGCAGCATATTTTACTGCTGGAGATTTTTGTAGATGTTACAAGTTCCGTGGAATGTTTGGAACTGCGAGATACTG |
| rs4986790 | GAAGGAAACTTGGAAAAGTTTGACAAATCTGCTCTAGAGGGCCTGTGCAATTTGACCATTGAAGAATTCCGATTAGCATACTTAGACTACTACCTCGATG[A/G]TATTATTGACTTATTTAATTGTTTGACAAATGTTTCTTCATTTTCCCTGGTGAGTGTGACTATTGAAAGGGTAAAAGACTTTTCTTATAATTTCGGATGG |
| rs7873784 | GAGGAAGGGAGAAATGAGGAAATAGGGAGTTGTCTAATTGGTATAAAATTATAGTATGCAAGATGAATTAGCTCTAAAGATCAGCTGTATAGCAGAGTTC[G/C]TATAATGAACAATACTGTATTATGCACTTAACATTTTGTTAAGAGGGTACCTCTCATGTTAAGTGTTCTTACCATATACATATACACAAGGAAGCTTTTG |
| rs2569190 | CTCCTCTGTGAACCCTGATCACCTCCCCACCTCTCTTCCTCCGAGCCAGCCCCCTTCCTTTCCTGGAAATATTGCAATGAAGGATGTTTCAGGGAGGGGG[A/G]CCGTAACAGGAAGGATTCTGCAGGGCATCTAGGGTTCTGTGTCTCCTGGCAGTGTCCTGATGACTCAGGCGCCCCAGGCGGTGAATGCCCTGTTGACTCG |
| rs4072037 | TACTCACAGCATTCTTCTCAGTAGAGCTGGGCACTGAACTTCTCTGGGTAGCCGAAGTCTCCTTTTCTCCACCTGGGGTAGAGCTTGCATGACCAGAACC[C/T]GTAACAACTGTTGCGGGTTTAGGGGCTGTGGTAGCTGTAAGAAGTTAAAGTCATAGGGTTGGGTCTTTATGAAGGAAAAATAAGAGCAAGAAGAAAATAT |
| rs11825977 | GGGCCAGGCTGAGGCCCCCGCCGGGGTGGARTCCATCCTGCTGACCATCAAGGATGACACCATCTACCTCACCCGCCACCTGGCTGTGCTTAACGGGGCC[G/A]TGTGAGTGTGGTCGGTGGCACCCCTCCCACATCCTAGCAACGGGGGCTGATGTTTCCCAAAGGGATATTCCTTGTAGCCCTAGAAGACCCCTTCCGCCCC |
| rs12008279 | GGAAGACAAGGTTATCAAGGGCATTGAATGTTGCATAGAGGTCATGGAAATTAAGGACTAAAAAGTGGCCATTGGATTTGGTGACATGCGGTCATTGGTA[A/G]CTGTGACAAGAACTTCTTTTGGTGGAACAGTGAGGGTGAAATGTCTCTAAGTCTCTCTCTGCCCTAGACCCTAACTCCTTCTCCCAGAGTCCATCCCTAC |
| rs12014762 | CGAAGTTTACCCCTCACTCAGCACTAGCCTGAAGAGTAAGTAGGTATATTCTGAGGGACATCAGATTCCACTCTAAAGTACTTCCCTAACTACCTGAAAT[C/T]CCATTAATTTGATGACAAATATATTTTTTAATTTGAGCCTTATAGGTTTTTCAAGAATCCCTTATTAAAATGCGAATACYAAAGCAGGCGAACAGATCAT |
| rs8629 | GCAAAGGTGCACTCTGCGAACGTTAAGTCCGTCCCCAGCGCTTGGAATCCTACGGCCCCCACAGCCGGATCCCCTCAGCCTTCCAGGTCCTCAACTCCCG[T/C]GGACGCTGAACAATGGCCTCCATGGGGCTACAGGTAATGGGCATCGCGCTGGCCGTCCTGGGCTGGCTGGCCGTCATGCTGTGCTGCGCGCTGCCCATGT |

**Supplementary Table 3.** Naïve regression models assessing the association between SNP genotype and PD risk

| **SNP & Genotype** | | APDR vs C | | PPMI vs C | |
| --- | --- | --- | --- | --- | --- |
|  |  | OR (95% CI) | *p* | OR (95% CI) | *p* |
| rs892145 | AA | - | - | - | - |
| PGLYRP2 | AT | 1.497 (1.041-2.152) | **.030*** | 1.125 (0.833-1.519) | .442 |
|  | TT | 1.638 (0.950-2.825) | .076 | 1.336 (0.843-2.118) | .218 |
| rs10888557 | GG | - | - | - | - |
| PGLYRP4 | GC | 1.644 (1.070-2.526) | **.023*** | 1.575 (1.085-2.288) | **.017*** |
|  | CC | 0.691 (0.138 – 3.461) | .653 | 0.578 (0.143-2.332) | .441 |
| rs4833095 | TT | - | - | - | - |
| TLR1 | TC | 0.855 (0.601-1.217) | .384 | 0.991 (0.736-1.334) | .951 |
|  | CC | 0.246 (0.084-0.717) | **.010*** | 0.791 (0.436-1.436) | .441 |
| rs3804099 | TT | - | - | - | - |
| TLR2 | TC | 0.895 (0.618-1.295) | .556 | 1.410 (1.016-1.956) | **.040*** |
|  | CC | 0.844 (0.514-1.387) | .504 | 1.553 (1.027-2.350) | **.037*** |
| rs4986790 | AA | - | - | - | - |
| TLR4 | AG | 0.594 (0.334-1.056) | .076 | 0.921 (0.601-1.411) | .705 |
|  | GG | NA^#^ | NA^#^ | 1.596 (0.265-9.612) | .610 |
| rs7873784 | GG | - | - | - | - |
| TLR4 | GC | 0.816 (0.550-1.211) | .314 | 1.137 (0.831-1.558) | .422 |
|  | CC | 2.110 (0.798-5.580) | .132 | 1.256 (0.478-3.305) | 0.644 |
| rs2569190 | GG | - | - | - | - |
| CD14 | GA | 0.641 (0.435-0.944) | **.024*** | 0.791 (0.567-1.103) | .167 |
|  | AA | 0.743 (0.465-1.185) | .212 | 0.808 (0.539-1.212) | .303 |
| rs4072037 | TT | - | - | - | - |
| MUC1 | TC | 1.025 (0.679-1.545) | .907 | 0.884 (0.633-1.235) | .471 |
|  | CC | 1.079 (0.662-1.757) | .761 | 0.827 (0.550-1.243) | .360 |
| rs11825977 | GG | - | - | - | - |
| MUC2 | GA | 0.964 (0.676-1.375) | .841 | 0.727 (0.535-0.988) | .**042*** |
|  | AA | 1.316 (0.548-3.160) | .538 | 0.895 (0.401-1.998) | .786 |
| rs12008279 | AA | - | - | - | - |
| CLDN2 | AG | 1.343 (0.853-2.114) | .203 | 1.201 (0.805-1.792) | .369 |
|  | GG | 1.030 (0.707-1.500) | .878 | 1.245 (0.912-1.701) | .168 |
| rs12014762 | CC | - | - | - | - |
| CLDN2 | CT | 1.437 (0.841-2.454) | .185 | 1.172 (0.731-1.879) | .511 |
|  | TT | 1.386 (0.857-2.241) | .183 | 1.180 (0.776-1.793) | .438 |
| rs8629 | CC | - | - | - | - |
| CLDN4 | CT | 0.885 (0.621-1.262) | .500 | 0.888 (0.661-1.192) | .428 |
|  | TT | 1.072 (0.597-1.926) | .816 | 0.633 (0.362-1.105) | .108 |

Major genotype is underlined. OR (95% CI), odds ratio (95% confidence interval). Bold values and *indicates p < 0.05 for OR compared to major genotype. #indicates unable to compute OR due to low sample number.

**Supplementary Table 4.** Generalised linear models investigating target SNPs and estimated mean age of PD symptom onset in PPMI cohort

| **Gene** | **SNP** | **GLMs in PPMI** | |
| --- | --- | --- | --- |
|  |  | **Naive** | **Corrected** |
| PGLYRP2 | rs892145 | 0.794 | 0.677 |
| PGLYRP4 | rs10888557 | 0.778 | 0.831 |
| TLR1 | rs4833095 | 0.350 | 0.371 |
| TLR2 | rs3804099 | 0.684 | 0.724 |
| TLR4 | rs4986790 | 0.443 | 0.463 |
| TLR4 | rs7873784 | 0.678 | 0.673 |
| CD14 | rs2569190 | 0.229 | 0.217 |
| MUC1 | rs4072037 | 0.672 | 0.600 |
| MUC2 | rs11825977 | 0.970 | 0.970 |
| CLDN2 | rs12008279 | 0.794 | 0.940 |
| CLDN2 | rs12014762 | 0.595 | 0.634 |
| CLDN4 | rs8629 | 0.204 | 0.257 |
